# Manufacture of a Polyaniline Nanofiber Ammonia Sensor Integrated with a Readout Circuit Using the CMOS-MEMS Technique

**DOI:** 10.3390/s90200869

**Published:** 2009-02-10

**Authors:** Mao-Chen Liu, Ching-Liang Dai, Chih-Hua Chan, Chyan-Chyi Wu

**Affiliations:** 1 Department of Mechanical Engineering, National Chung Hsing University, Taichung, 402 Taiwan, ROC; E-Mails: d9361301@mail.nchu.edu.tw; libra_louis@hotmail.com; 2 Department of Mechanical and Electro-Mechanical Engineering, Tamkang University, Tamsui, 251 Taiwan, ROC; E-Mail: ccwu@mail.tku.edu.tw

**Keywords:** Ammonia sensor, CMOS, Polyaniline, readout circuit

## Abstract

This study presents the fabrication of a polyaniline nanofiber ammonia sensor integrated with a readout circuit on a chip using the commercial 0.35 μm complementary metal oxide semiconductor (CMOS) process and a post-process. The micro ammonia sensor consists of a sensing resistor and an ammonia sensing film. Polyaniline prepared by a chemical polymerization method was adopted as the ammonia sensing film. The fabrication of the ammonia sensor needs a post-process to etch the sacrificial layers and to expose the sensing resistor, and then the ammonia sensing film is coated on the sensing resistor. The ammonia sensor, which is of resistive type, changes its resistance when the sensing film adsorbs or desorbs ammonia gas. A readout circuit is employed to convert the resistance of the ammonia sensor into the voltage output. Experimental results show that the sensitivity of the ammonia sensor is about 0.88 mV/ppm at room temperature.

## 1. Introduction

Ammonia (NH_3_) is emitted by human activity. For instance, production of refrigeration systems and fertilizers emits ammonia. The worldwide ammonia emission resulting from domestic animals and industrial combustion is about 20-35 Tg/year and 2.1-8.1 Tg/year, respectively [1]. Ammonia is a toxic gas with exposure limit values of 25 ppm for a period of 8 h and of 35 ppm for a period of 10 min [2]. Ammonia sensors are important devices in many industrial, agricultural and biomedical applications. For instance, Boeker *et al.* [3] proposed an ammonia sensor based on a quartz crystal microbalance transducer for monitoring the ammonia concentration in agricultural emissions. Moos *et al.* [4] developed an ammonia gas sensor with a selective thick film of zeolites for automotive exhaust gas applications. Pushkarsky *et al.* [5] presented a laser-based photoacoustic ammonia sensor for industrial ammonia gas measurement.

Huang *et al.* [6] developed polyaniline nanofibers using interfacial polymerization at an aqueous and organic interface, and the resulting polyaniline, which had high surface area and porosity, could enhance properties in applications such as chemical sensors. With the same synthetic method, Virji *et al.* [7] fabricated polyaniline nanofiber gas sensors with performances that were better than conventional polyaniline. Thereby, the ammonia sensor in this work adopts polyaniline as a sensitive material. Microelectromechanical systems (MEMS) technology was applied to fabricate various microsensors. The advantages of microsensors include small size, high performance, low cost and easy mass-production. Many studies have recently utilized MEMS technology to manufacture ammonia sensors. For instance, Lee *et al.* [8] fabricated a micro ammonia gas sensor using a bulk micromachining technique. The sensor that was resistive type composed of a polyaniline film, a SU-8 adhesion layer and an interdigital Pt electrode, where the polyaniline film was the ammonia sensing film. The ammonia sensor had a sensitivity of about 40% at 50 ppm ammonia. Mitzner *et al.* [9] utilized a post-CMOS process to manufacture a micro-hotplate-based gas sensor array. The post-CMOS process adopted XeF_2_ to etch a silicon substrate to obtain the micro-hotplate array, and a SnO_2_/Pt sensing film was sputtered on this micro-hotplate forming a resistive-type ammonia sensor. Llobet *et al.* [10] utilized silicon process technology to make an ammonia sensor, in which the sensor consisted of a heater, an interdigital electrode, a temperature meter and a sensing film. Tungsten oxide, coated on the interdigital electrode, was adopted as an ammonia sensing film. Li *et al.* [11] presented a micro gas with piezoresistive SiO_2_ cantilever beam fabricated by a surface and bulk micromachining process. An ammonia sensing layer of 11-mercaaptoundecanoic acid was coated on the piezoresistive cantilever beams. The sensor was packaged with a linear amplifier, and its output voltage was about 7 μV in 1 ppm ammonia gas. Connolly *et al.* [12] reported a porous SiC ammonia sensor produced by silicon process technology, in which the sensor was a capacitive type. The sensing film of SiC was deposited by plasma enhanced chemical vapor deposition (PECVD) and was then made porous by electrochemical etching in 73% HF.

The use of a commercial CMOS process to fabricate MEMS devices is known as the CMOS-MEMS technique [13]. Several microdevices manufactured by the CMOS-MEMS technique need a post-process to coat the functional films or release the suspended structures. For instance, the suspended structures of the CMOS-MEMS switches [14] and pressure sensors [15] were released by a wet etching post-process. The main advantage of microdevices fabricated by the CMOS-MEMS technique is their ability to integrate with circuits as a system on a chip (SOC) because of their compatibility with the CMOS process. The ammonia sensors, proposed by Lee *et al.* [8], Mitzner *et al.* [9], Llobet *et al.* [10], Li *et al.* [11] and Connolly *et al.* [12], had no integration with circuitry on a chip. In this work, we employ the CMOS-MEMS technique to fabricate a polyaniline nanofiber ammonia sensor integrated with a readout circuit on a chip, which can reduce the volume and cost and enhance the performance. The area of the integrated ammonia sensor chip is about 0.6 mm^2^.

## 2. Structure of the Integrated Ammonia Sensor

**Figure 1.**
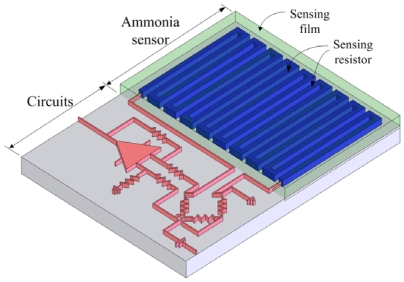
Schematic structure of the ammonia sensor integrated with readout circuit.

Figure 1 shows the structure of the integrated chip, which contains an ammonia sensor and readout circuit. The ammonia sensor is composed of a sensing resistor and an ammonia sensing film. The sensing resistor is a polysilicon winding line. The ammonia sensing film, which is polyaniline prepared by a chemical polymerization method, is coated on the sensing resistor. A silicon dioxide layer is located between the sensing resistor and the polyaniline film. The sensing resistor is 2 μm wide, 0.4 μm thick and 9,000 μm long.

**Figure 2.**
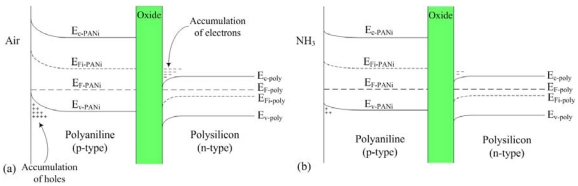
Energy band diagram of the sensor in **(a)** air and **(b)** ammonia. E_F_ is the Fermi level, E_c-PANi_ is the conduction band of polyaniline, E_v- PANi_ is the valence band of polyaniline, E_Fi-PANi_ is the intrinsic Fermi level of polyaniline, E_c-PolySi_ is the conduction band of polysilicon, E_v-PolySi_ is the valence band of polysilicon, and E_Fi-PolySi_ is the intrinsic Fermi of polysilicon.

Figure 2 illustrates the energy band diagram of the ammonia sensor. When the polyaniline surface contacts air, holes are produced on this surface. As shown in Figure 2(a), an accumulation of holes is formed on the surface of the polyaniline film, so that the valence band edge of polyaniline bends upward and is close to the Fermi level of polyaniline. An accumulation of electrons at the oxide-polysilicon interface is formed [16], which causes the conduction band edge of polysilicon to bend downward and be close to the Fermi level of polysilicon. When polyaniline interacts with ammonia, the following reaction occurs [17]:

(1) $${\text{PANIH}}^{+}+{\text{NH}}_{3}↔\text{PANI}+{\text{NH}}_{4}^{+}$$

According to Equation (1), the H^+^ holes of polyaniline reduce as polyaniline interacts with NH_3_. As shown in 2(b), the accumulation of holes at the surface of polyaniline is reduced when polyaniline is exposed to ammonia, resulting in the valence band edge of reduced polyaniline bending and having a distance with the Fermi level of polyaniline, so that the resistance of polyaniline increases. At the same time, the accumulation of electrons at the oxide-polysilicon interface is reduced, which leads to the conduction band edge of polysilicon to reduce bending and have a distance with the Fermi level of polysilicon, so that the resistance of polysilicon increases.

**Figure 3.**
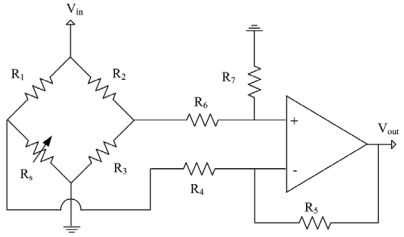
Readout circuit for the ammonia sensor.

**Figure 4.**
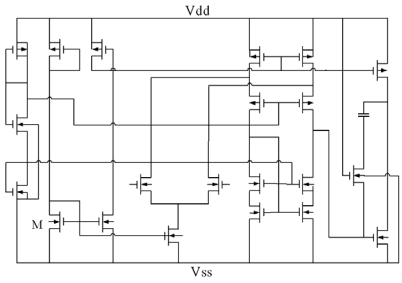
Design of the operational amplifier circuit.

Figure 3 presents the readout circuit for the ammonia sensor. The readout circuit consists of a Wheatstone circuit, an operational amplifier and resistances. As shown in Figure 3, the Wheatstone circuit is constituted by the sensing resistor (*R_s_*) and three resistances (*R_1_, R_2_* and *R_3_*). The sensing resistor changes its resistance as the sensing film adsorbs or desorbs ammonia gas. The readout circuit is utilized to convert the resistance of the ammonia sensor into the voltage output. In this design, *R_1_*=10 kΩ, *R_2_*=10 kΩ, *R_3_*=10 kΩ, *R_4_*=100 Ω, *R_5_*=10 kΩ, *R_6_*=10 kΩ and *R_7_*=10 kΩ are adopted. Figure 4 illustrates the operational amplifier circuitry for the readout circuit, where *V_dd_* represents a voltage power supply and *Vss* is the ground.

**Figure 5.**
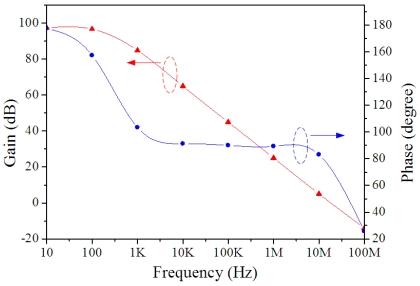
Frequency response of the operational amplifier.

HSPICE, which is a professional circuit simulation software, was used to simulate the operational amplifier and the readout circuit. Figure 5 shows the simulated results of the frequency response for the operational amplifier. As shown, the operational amplifier has a dc open loop gain of approximately 97 dB.

**Figure 6.**
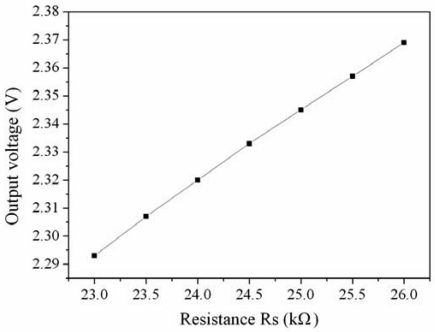
Simulated results of the readout circuit.

Figure 6 depicts the simulated results of the readout circuit. In this simulation, the input voltage *V_in_* is 3 V, the resistance of the ammonia sensor *R_s_* varies from 23 kΩ to 26 kΩ. The output voltage of the readout circuit varies from 2.294 V to 2.372 V as the resistance of the ammonia sensor changes from 23 kΩ to 26 kΩ.

## 3. Fabrication of the Integrated Ammonia Sensor

The commercial 0.35 μm CMOS process of Taiwan Semiconductor Manufacturing Company (TSMC) was used to fabricate the integrated chip with ammonia sensor and readout circuit. In order to expose the sensing resistor and coat the ammonia sensing film, the integrated chip requires a post-process after completion of the CMOS process. The post-process contains two main steps: (1) etching the sacrificial layers to expose the sensing resistor, and (2) coating the ammonia sensing film on the sensing resistor.

**Figure 7.**
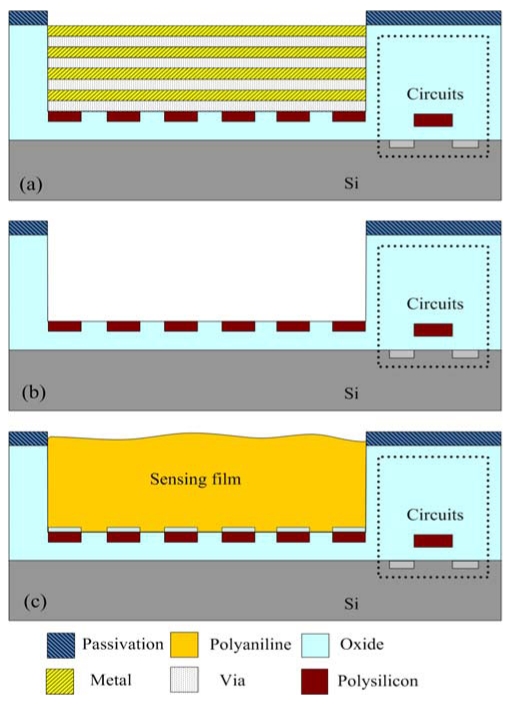
Process flow of the ammonia sensor; (a) after the CMOS process, (b) etching sacrificial layers, and (c) coating the sensing film.

**Figure 8.**
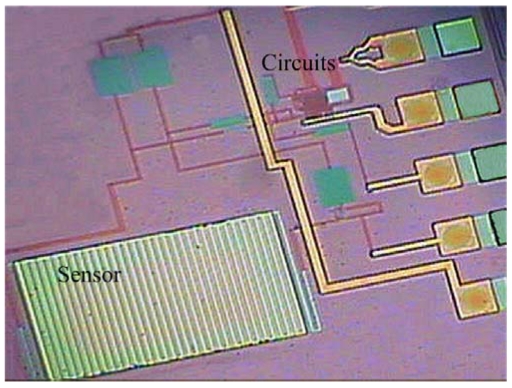
photograph of the integrated ammonia sensor chip after the wet etching process.

Figure 7 shows the process flow of the integrated chip. Figure 7(a) illustrates the cross-section of the integrated chip after completion of the CMOS process. In the ammonia sensor, the polysilicon layer is adopted as the sensing resistor, and the metal and via layers are used as the sacrificial layers. The materials of the metal and via layers are aluminum (Al) and tungsten (W), respectively. The sacrificial layers are removed from the ammonia sensor, exposing the sensing resistor. As shown in Figure 7(b), the chip is immersed in two etchants: one is an Al etchant with phosphoric acid, nitric acid, acetic acid and deionized water in a ratio of 14:1:2:3. The other is a W etchant with sulfuric acid and hydrogen peroxide in a ratio of 2:1. Figure 8 displays the photograph of the integrated ammonia sensor after the wet etching process. Then, the chip is put in an oven at 300°C for 8 h, so that a thin silicon dioxide layer is formed on the polysilicon surface. Finally, the polyaniline film is coated on the sensing resistor as shown in Figure 7(c).

The polyaniline nanofibers [6,18] are prepared by a chemical polymerization method according to the following steps: (1) 8 mL HCl is mixed with 100 mL deionized water and stirred until a homogenous solution is obtained; (2) the solution of 1 mL C_6_H_5_NH_2_ is added into the HCl solution with stirring until the mixing solution becomes homogenous; (3) 2.4 g (NH_4_)_2_S_2_O_8_ is dissolved in 50 mL deionized water by stirring vigorously; (4) the (NH_4_)_2_S_2_O_8_ solution is added into the C_6_H_5_NH_2_/HCl solution and stirred until a homogeneous mixing solution is obtaned; (5) the mixing solution of (NH_4_)_2_S_2_O_8_/C_6_H_5_NH_2_/HCl is aged at room temperature for 120 h, producing a blackish green sediment; (6) the resulting product is filtered, and a precision micro dropper is used to drop the product on the ammonia sensor chip, followed by calcination in air at 110°C for 1 h.

**Figure 9.**
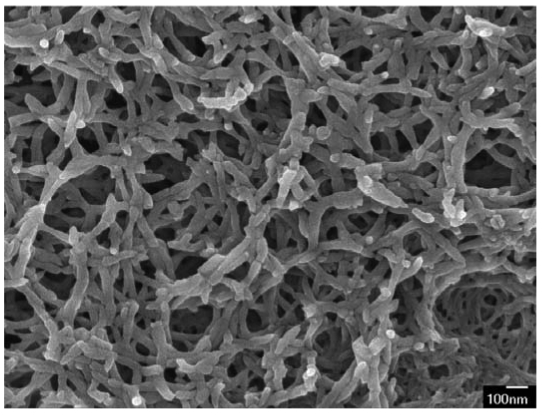
Scanning electron microscope image of polyaniline nanofiber film.

**Figure 10.**
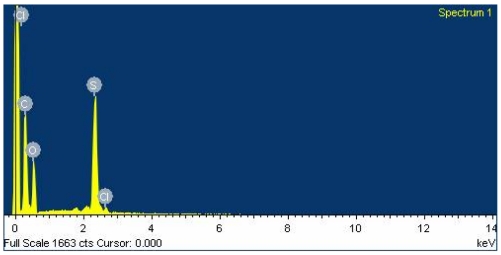
Elements of polyaniline nanofiber film measured by energy dispersive spectrometer.

The surface morphology of the polyaniline film is measured by a scanning electron microscope (JEOL JSM-6700F). Figure 9 shows a scanning electron microscope image of the polyaniline film. The sensing film, which is composed of many polyaniline nanofibers as shown in Figure 9, is porous and has a large surface area. The elements of the polyaniline nanofiber film are measured by an energy dispersive spectrometer (OXFORD INCA ENERGY 400), and the measured results are shown in Figure 10. The polyaniline nanofiber film contains 63 wt% C, 28 wt% O, 8.5 wt% S and 0.5 wt% Cl.

## 4. Results and Discussion

**Figure 11.**
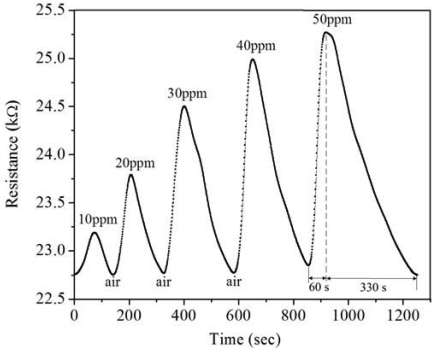
Relation between the resistance variation and NH_3_ concentration for the ammonia sensor.

A power supply, an oscilloscope, an LCR meter and a test chamber were utilized to measure the performance of the ammonia sensor chip. In order to characterize the variation of resistance in the sensing part, the ammonia sensor was tested without readout circuit. The sensor chip was set in the test chamber, and the LCR meter was used to measure its resistance variation at different NH_3_ concentrations. Figure 11 presents the measured results of the ammonia sensor without readout circuit at different ammonia concentrations. The initial resistance of the sensing resistor in the ammonia sensor was about 22.7 kΩ (in air), and the resistance of the sensor varied to 25.5 kΩ at 50 ppm NH_3_. The results showed that the resistance of the ammonia sensor increased as the concentration of ammonia increased. As shown in Figure 11, the ammonia sensor had a response time of about 60 sec at 50 ppm NH_3_ and a recovery time of 330 sec at 50 ppm NH_3_.

**Figure 12.**
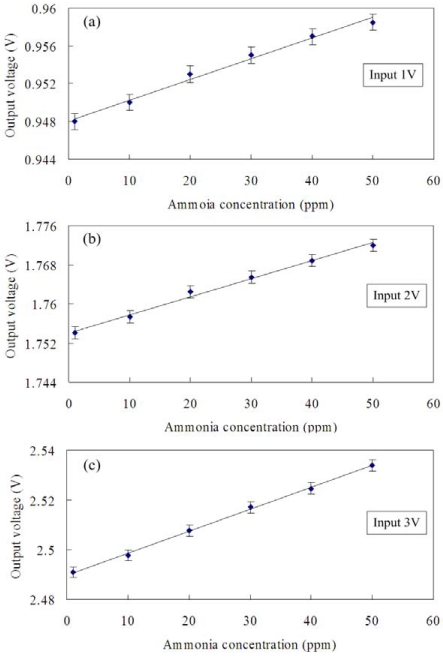
Measured results of the ammonia sensor with input voltage of (a) 1 V, (b) 2 V and (c) 3 V.

The ammonia sensor with readout circuit was set in the test chamber and was tested at room temperature with different ammonia concentrations. In order to understand the influence of the input voltage, the sensor was provided with different input voltages. The power supply provided a bias voltage of 3.3 V and different input voltages of 1, 2 and 3 V, respectively, to the readout circuit in the sensor. The oscilloscope was employed to record the output signal of the sensor to ammonia changes. Figure 12 displays the output voltage of the ammonia sensor with different input voltages of 1, 2 and 3 V. In this investigation, 1 to 50 ppm of ammonia gas was supplied. With an input voltage of 1 V, the output voltage of the ammonia sensor changed from 948 mV to 958 mV as the concentration of ammonia gas varied from 1 to 50 ppm. The variation of the output voltage was 10 mV over 1-50 ppm NH_3_, so the sensitivity of the ammonia sensor with an input voltage of 1 V was about 0.2 mV/ppm. As shown in Figure 12(b), when an input voltage of 2 V was supplied, the measured results showed that the output voltage of the ammonia sensor varied from 1.754 V to 1.772 V as the concentration of ammonia gas changed from 1 to 50 ppm. Thereby, the variation of the output voltage was 18 mV in 1-50 ppm NH_3_, and the ammonia sensor with an input voltage of 2 V had a sensitivity of about 0.36 mV/ppm. When the input voltage was changed to 3 V, as depicted in Figure 12(c), the output voltage of the ammonia sensor changed from 2.491 V to 2.534 V as the concentration of ammonia gas varied from 1 to 50 ppm. The variation of the output voltage was 43 mV for 1-50 ppm NH_3_, so the sensitivity of the ammonia sensor with an input voltage of 3 V was approximately 0.88 mV/ppm. Therefore, the integrated ammonia senor had a sensitivity of 0.88 mV/ppm when providing a bias voltage of 3.3 V and an input voltage of 3V. The polyaniline nanofiber gas sensor, fabracted by Virji *et al.* [7], was used to detect a low concentration of 3 ppm ammonia gas and had a high sensitivity. Sutar *et al.* [19] proposed a polyaniline nanofiber ammonia sensor with the detection limit of 0.5 ppm NH_3_. Crowley *et al.* [20] manufactured an ammonia gas sensor by using inkjet-printed polyaniline nanoparticles, and the sensor had a detection limit of 1 ppm NH_3_. In this study, the experiments showed that the detection limit of the ammonia sensor was about 1 ppm NH_3_. The resistive ammonia sensor with a sensitive film of polyaniline, presented by Lee *et al.* [8], had a sensitivity of about 40% at 50 ppm ammonia. Li *et al.* [11] proposed an ammonia sensor with a sensitive material of 11-mercaaptoundecanoic acid, and it had an output voltage of 7 μV in 1 ppm ammonia gas. The sensitivity of 0.88 mV/ppm in this work exceeds that of Li *et al.* [11].

## 5. Conclusions

A polyaniline nanofiber ammonia sensor integrated with readout circuit was successfully implemented using the commercial 0.35 μm CMOS process and the post-process. The ammonia sensor was comprised of a sensing film and a sensing resistor. The sensing film, which was prepared by a chemical polymerization method, was composed of polyaniline nanofibers that had a large surface area. The sensing resistor was formed by the polysilicon layer of the CMOS process. The post-process used etchants to etch the sacrificial layers to expose the sensing resistor, and then polyaniline was coated on the sensing resistor. The resistance of the resistive type ammonia sensor changed upon ammonia gas adsorption. A readout circuit was employed to convert the variation in resistance of the ammonia sensor into a voltage output. Experimental results showed that the sensitivity of the ammonia sensor was about 0.88 mV/ppm at room temperature when providing a bias voltage of 3.3 V and an input voltage of 3 V.
